# Towards Conservation of the Remarkably High Number of Daisy Trees (Asteraceae) in Mexico

**DOI:** 10.3390/plants10030534

**Published:** 2021-03-12

**Authors:** Rosario Redonda-Martínez, Patricio Pliscoff, Andrés Moreira-Muñoz, Esteban Manuel Martínez Salas, Marie-Stéphanie Samain

**Affiliations:** 1 Instituto de Ecología, A.C., Red de Diversidad Biológica del Occidente Mexicano, Pátzcuaro 61600, Michoacán, Mexico; mariestephanie.samain@gmail.com; 2 Departamento de Ecología, Facultad de Ciencias Biológicas, Pontificia Universidad Católica de Chile, Alameda 340, Santiago 8331150, Chile; pliscoff@uc.cl; 3 Instituto de Geografía, Facultad de Historia, Pontificia Universidad Católica de Chile, Geografía y Ciencia Política, Avenida Vicuña Mackenna 4860, Macul, Santiago 7820436, Chile; 4 Center of Applied Ecology and Sustainability (CAPES), Pontificia Universidad Católica de Chile, Santiago 8331150, Chile; 5 Instituto de Geografía, Facultad de Ciencias del Mar y Geografía, Pontificia Universidad Católica de Valparaíso, Avenida Brasil 2241, Valparaíso 2340000, Chile; andres.moreira@pucv.cl; 6 Departamento de Botánica, Instituto de Biología, Universidad Nacional Autónoma de México, Herbario Nacional de México, Mexico City 04510, Mexico; ems@ib.unam.mx

**Keywords:** biogeographic provinces, Compositae, endemism, nectariferous plants, ornamental species, protected areas, species distribution modelling, traditional medicine

## Abstract

Mexico is floristically the fourth most species-rich country in the world, and Asteraceae is the most diverse vascular plant family in this country. The species exhibits a wide range of growth forms, but the tree-like habit, appropriately named daisy trees, is heavily underestimated, even though slightly different tree definitions are handled. Very little is known about their precise species number or conservation status in Mexico, so we update here the list of known Mexican daisy tree species, summarize their very diverse uses, present a general panorama of their present and future distribution, and discuss their conservation status. A bibliographic review and herbarium study were carried out, carefully curated taxonomical ocurrence maps were prepared for each species, and a climatic suitability modelling approach was used to characterise the spatial patterns of Mexican Asteraceae trees. With 149 daisy tree species, the country ranks second at a global level; within the country, their greatest diversity is found in central and western Mexico. A decrease in diversity is estimated in areas that currently host the highest species richness, whereas the hotspot regions are estimated to show an increase in species diversity, so climate change is not a threat to all Mexican daisy tree species.

## 1. Introduction

With more than 23,000 vascular plant species, Mexico is floristically the fourth most species-rich country in the world, after Brazil, China, and Colombia [1,2]; 11,600 of the Mexican plant species are endemic [1]. Asteraceae is the most diverse family of vascular plants in Mexico, with 417 genera and 3050 native species, of which 1988 are endemic, representing about 65% of the family in Mexico [3]. In the flora of North America, 418 genera and 2413 species of this family are registered [4]; in Brazil, 310 genera (64 endemic, 17 exotic) and 2113 species (42 introduced) are registered [5]; in the flora of China, 248 genera (18 endemic, 49 introduced) and 2336 species (1145 endemic, 109 introduced) are registered [6]; in Colombia, 258 genera and 1302 species are registered [7]; while in Ecuador, 217 genera and 918 species (360 endemic) are registered [8]. Its representatives are found practically everywhere on the planet, except in Antarctica and polar regions with permanent ice [9,10]. In Mexico, they are distributed from sea level in coastal dunes to the alpine grasslands of mountainous regions at more than 4000 m elevation [3].

Asteraceae is characterized by its inflorescences called head or capitulum, that simulate a flower that contains numerous florets with unilocular, bicarpellate, inferior ovary, and syngeneic stamens. Their diversity and distribution are due, amongst others, to effective dispersal mechanisms of its fruits by the pappus, the modified calyx (in some cases, the apex of the cypsela lengthens, forming a hook-like structure, as, e.g., in dandelion, *Taraxacum officinale*, and several Mutisieae, which functions as an aerodynamic structure similar to a propeller to disperse the fruits with the help of the wind), and short life cycles in most of its members. The latter characteristic allows them to colonize disturbed environments or sites where the original vegetation has been removed, thus being essential elements of secondary vegetation, ruderal and weeds in various crops [10,11].

**Figure 1:**
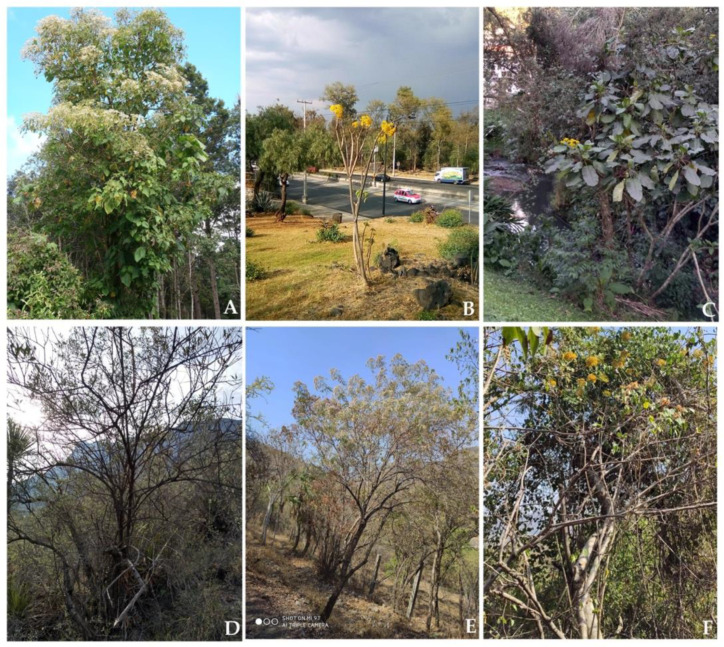
Selection of Mexican arborescent species of Asteraceae. (**A**) *Montanoa hexagona* (Heliantheae), (**B**) *Pittocaulon praecox* (Senecioneae), (**C**). *Telanthophora grandiflora* (Senecioneae), (**D**) *Nahuatlea smithii* (Gochnatieae), (**E**) *Critoniopsis uniflora* (Vernonieae), (**F**) *Sinclairia glabra* (Liabeae). Photo credits: (**A**–**D**) Rosario Redonda-Martínez; (**E**–**F**) Fernando Araujo-Mondragón.

Members of the Asteraceae exhibit a wide range of growth forms, including short-lived annual or perennial herbs, subshrubs, shrubs, trees, and even climbing, epiphytic and (sub)aquatic plants [10]. In the particular case of trees, there are some studies dealing with Mexican species. Standley [12] was one of the first to document the diversity of woody species in Mexico; in the case of Asteraceae, he recorded mainly shrubs and only 14 tree or tree-like species. Other studies where tree species have been included correspond to the taxonomic reviews of some tribes [13,14], genera [15,16,17,18,19,20,21], or sections of these [22,23]. The most recent publications that include arborescent Asteraceae [24,25,26] consider only 36, 62, and 41 species, respectively. Even though at least some of these discrepancies might be due to different tree definitions applied, we consider that a considerable cause is what we might call “Asteraceae tree blindness”; most people, including botanists, picture representatives of this family as annual herbs or short-lived perennials, contrasting with the surprisingly high number of woody species it contains. These trees with their beautiful and striking inflorescences are appropriately called daisy trees (Figure 1).

Very little is known about the conservation status of Mexican Asteraceae species in general, and of tree species of this family in particular. Therefore, within the framework of the Global Tree Assessment, in cooperation with the IUCN/SSC Global Tree Specialist Group and Botanic Gardens Conservation International, all arborescent Asteraceae species that are endemic or near-endemic to Mexico (i.e., those shared with the south of the United States of America north of Mexico, and those shared with Central America south of the country) are being assessed for the IUCN Red List. Therefore, we use here the tree definition agreed on by the IUCN/SSC Global Tree Specialist Group, which has also been applied by [25]: a woody plant, usually with a single stem growing to a height of at least 2 m, or if multi-stemmed, then at least one vertical stem 5 cm in diameter at breast height.

A recent exploratory study, including species distribution and spatial analyses of a comprehensive list of native Mexican trees, carried out by [26], included 41 arborescent Asteraceae species. However, based on our knowledge of Asteraceae on the one hand, and our ongoing red listing work on the other hand, we realized that (1) this number is heavily underestimated, even though we handle a slightly different tree definition, and (2) the data analyzed were obtained from the National Biodiversity Information System database of Mexico [27] which, although it compiles and georeferences information, has insufficient taxonomic curation. Moreover, during the preparation of our Red List assessments, we noticed that information on arborescent Asteraceae, as is also the case for tree species in general, is very scattered and knowledge quite limited. As a consequence, a first step in the conservation of these species is the compilation of relevant information in order to obtain a general overview of their distribution, threats, and conservation status.

Based on our ongoing Red List assessments of endemic and near-endemic Mexican Asteraceae and a meta-analysis of carefully curated distribution data, the objectives of this study are the following: (1) to document the precise number of Asteraceae trees that are distributed in Mexico and update the list of Mexican arborescent Asteraceae; (2) to summarize their very diverse uses; (3) to present a general panorama of their present and future distribution, including characterization of climatic suitability; and (4) to discuss the impact on their conservation in protected areas and biogeographical provinces.

## 2. Results

### 2.1. Species List

The list, generated from the bibliographic review and study of herbarium specimens, includes 149 tree species of Asteraceae, distributed in three subfamilies and 12 tribes (Appendix A), with Asteroideae having 129 species, being the most diverse. The latter subfamily consists of the tribes Heliantheae (54 species), Eupatorieae (42), and Senecioneae (20), which contain the highest number of species, whereas the remaining six tribes are each represented by only one to four species. Following in order of importance, there is subfamily Vernonioideae, in which the tribe Vernonieae groups 16 taxa and Liabeae only one. Finally, the subfamily Gochnatioideae represented by the Gochnatieae tribe includes only three species (Appendix A, Table 1). At the tribe level, Eupatorieae, Heliantheae, Senecioneae, and Vernonieae represent about 89%, while the other eight are equivalent to the remaining 11% of the total number of daisy trees.

Some species reported as arborescent both in the literature and on the labels of herbarium specimens were excluded from the list because they have been synonimized, e.g., *Roldana cordovensis*.

### 2.2. Uses of Daisy Trees

Of the 149 daisy tree species, just under 50% have a registered use. Of the 65 potentially used species, 37 have medicinal purposes, the leaves or young branches being the most used parts. Regarding the diseases they cure or the healing properties attributed to them, 12 species stand out as anti-inflammatory, 11 are used to treat stomach diseases, and 10 for skin conditions, followed by five used as antiseptics and five as febrifugals; they are also used to treat oral, heart, kidney, rheumatism, and vertigo conditions. Moreover, eight species with various medicinal uses were recorded.

Their usefulness as nectariferous species also stands out, distinguishing two main groups of insects and a group of birds, for which they serve as food for honeybees (*Apis mellifera*), butterflies, and hummingbirds, with 17, 3, and 1 species, respectively.

Six species are applied as forage, and of these, four are used only for that purpose, mainly when they are found in arid or semi-arid zones. Other documented uses for Asteraceae trees are as a living fence, cut flower, artisanal, ceremonial, fuel, construction, insecticide, ritual, and shade for coffee [29] (Table 2).

### 2.3. Diversity per Vegetation Type

The Mexican daisy trees occur in practically all vegetation types and most grow in several vegetation types, although the majority show an affinity for temperate and humid environments. Hence, the highest number of species are recorded in pine forests (107 spp.), followed by oak forests (104 spp.) and cloud forests (90 spp.). However, dry areas also host an important diversity, as 85 species are found in low deciduous forests and 47 in crassicaule shrubland. It should be noted that the genus *Nahuatlea* is exclusively distributed in arid and semi-arid areas of Mexico, being an important part of the vegetation structure in the crassicaule and thorny shrublands of the south and central part of the country. Finally, disturbed sites also host a significant number of arborescent Asteraceae, with 86 species, thus demonstrating the importance of this family as dominant elements of secondary vegetation [29].

### 2.4. Distribution in Mexico

**Figure 2:**
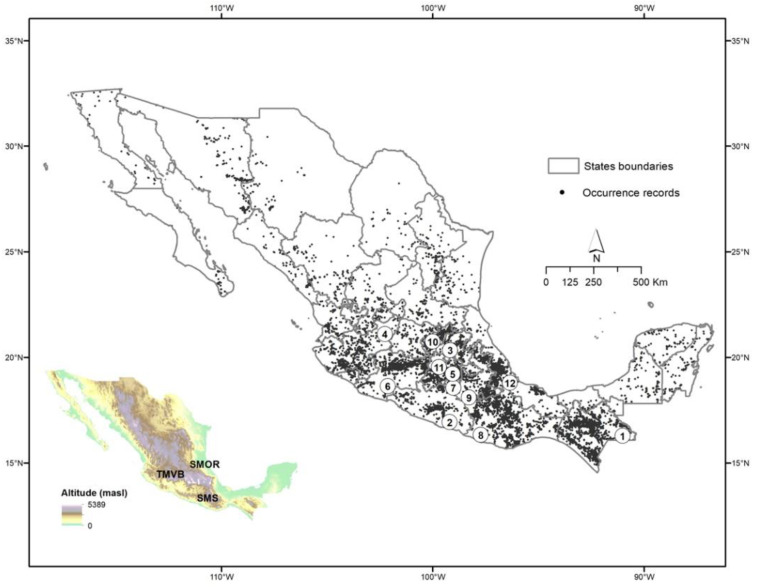
Occurrence records of Asteraceae tree species in Mexico. The states with the highest number of species are the following: 1 Chiapas, 2. Guerrero, 3. Hidalgo, 4. Jalisco, 5. Mexico City, 6. Michoacán, 7. Morelos, 8. Oaxaca, 9. Puebla, 10. Querétaro, 11. State of Mexico, 12. Veracruz. The map at the left shows the principal mountain regions mentioned in the text: Trans-Mexican Volcanic Belt (TMVB), Sierra Madre del Sur (SMS) and Sierra Madre Oriental (SMOr).

Asteraceae trees are found in almost the entire territory; however, the highest number of species is found in the center and south of the country, mainly in the Trans-Mexican Volcanic Belt (Hidalgo, Jalisco, Mexico City, Michoacán, Morelos, State of Mexico, Puebla, Veracruz), the Sierra Madre del Sur (Chiapas, Guerrero, Oaxaca) and the southern portion of the Sierra Madre Oriental (Puebla, Querétaro, Veracruz). (Figure 2).

### 2.5. Climatic Suitability Patterns

**Figure 3:**
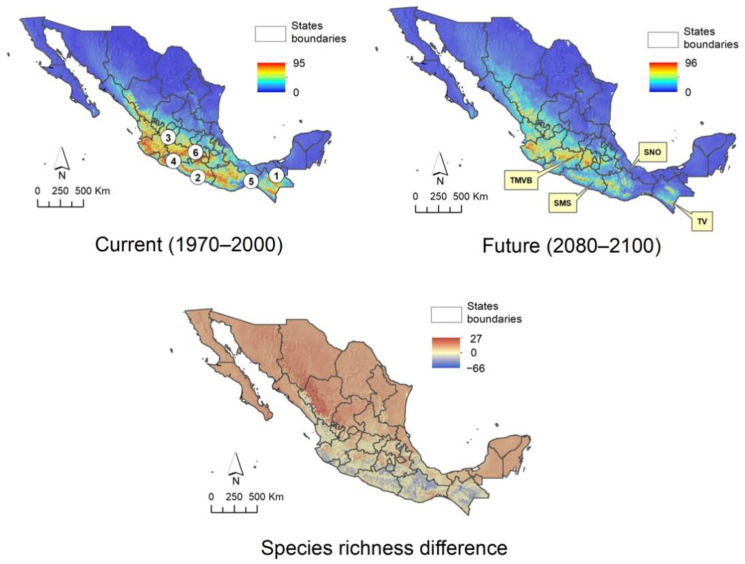
Effect of climate change on Mexican Asteraceae tree species in two different scenarios: current (1970 to 2000) and future (2080 to 2100) and the difference between both. States with the highest species richness: 1. Chiapas, 2. Guerrero, 3. Jalisco. 4. Michoacán, 5. Oaxaca, 6. State of Mexico. Regions identified as Anthropocene refugia: Trans-Mexican Volcanic Belt (TMVB), Sierra Madre del Sur (SMS), Sierra Norte de Oaxaca (SNO), and Tacaná Volcano (TV).

The climatic suitability patterns of Asteraceae tree species in Mexico were characterized using models of 86 species, 17 of which show an expansion of over 10% of their current range, whereas 33 species exhibit a contraction of over 50% of their current range; both cases occurs under future scenario (Appendix B). Figure 3 depicts the current and future climatic suitability patterns in Mexico and the difference between scenarios. In the current scenario (1970 to 2000), it is clearly observed that the greatest diversity is found in the west, center, and south of the country, with the states of Jalisco, Michoacán, Mexico, Guerrero, Oaxaca, and Chiapas being those that host the greatest diversity of daisy tree species. The future model (2080 to 2100) estimates a drastic decrease in the number of species in the aforementioned states, although it is more noticeable in Oaxaca. As can be seen on the map that summarizes current and future differences, this state, together with Guerrero, Chiapas, and Jalisco, are those that are estimated to lose the greatest diversity. However, the results also show that the mountain regions of Guerrero and Oaxaca belonging to the Sierra Madre del Sur (SMS) and the Sierra Norte de Oaxaca (SNO), the Tacaná Volcano (TV) in Chiapas on the border with Guatemala, the south of the State of Mexico, northern Michoacán, and the western portion of Jalisco, corresponding to the Trans-Mexican Volcanic Belt (TMVB), will maintain a considerable diversity, indicating that these areas could function as Anthropocene refugia for daisy trees.

### 2.6. Protected Area Network and Biogeographic Provinces

**Figure 4:**
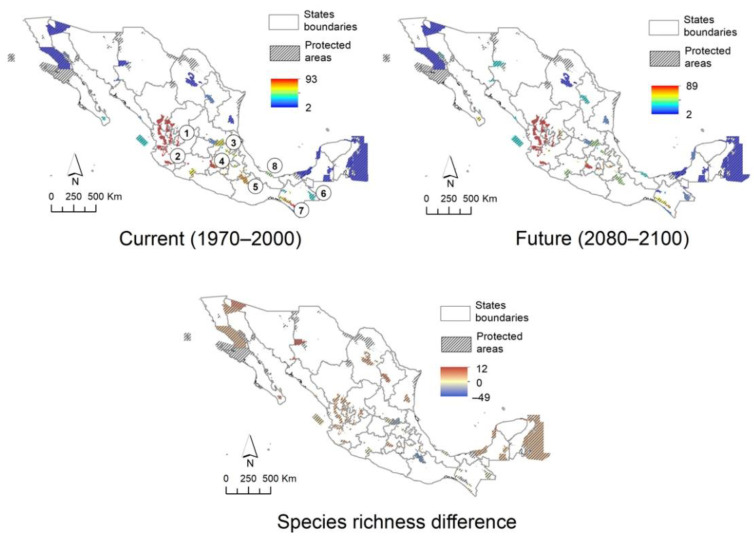
Actual and future scenarios for Mexican Asteraceae tree richness in the system of Natural Protected Areas at federal level. 1. Flora and Fauna Protection Area “Cuenca Alimentadora del Distrito Nacional de Riego 043, Estado de Nayarit”, 2–8. Biosphere Reserves. 2 Sierra de Manantlán, 3. Sierra Gorda, 4. Monarch Butterfly, 5. Tehuacán-Cuicatlán Valley, 6. Montes Azules, 7. El Triunfo, 8. Los Tuxtlas.

**Figure 5:**
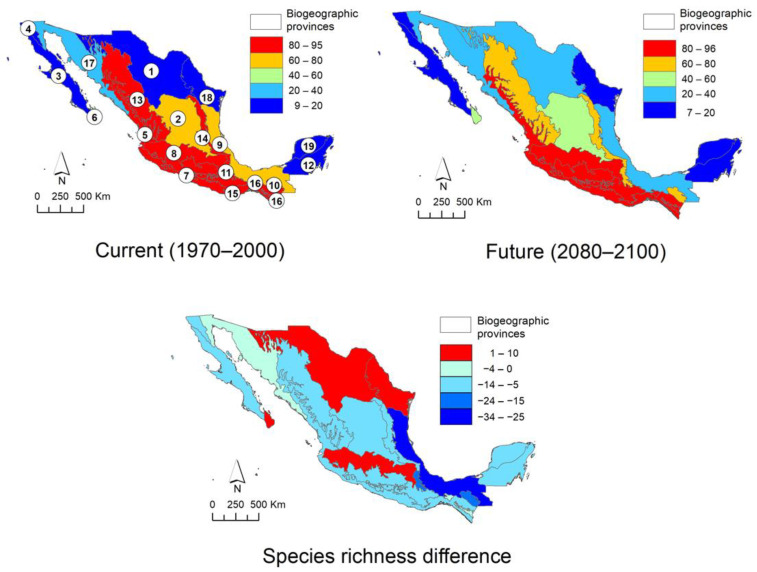
Estimation of the changes in the distribution of Mexican Asteraceae trees in the different biogeographical provinces of Mexico. 1. Northern Altiplano (Chihuahuense), 2. Southern Altiplano (Zacatecano–Potosina), 3. Baja California, 4. California, 5. Pacific Coast, 6. Cape, 7. Balsas Depression, 8. Trans-Mexican Volcanic Belt, 9. Gulf of Mexico, 10. Altos de Chiapas, 11. Oaxaca, 12. Petén, 13. Sierra Madre Occidental, 14. Sierra Madre Oriental, 15. Sierra Madre del Sur, 16. Soconusco, 17. Sonorense, 18. Tamaulipeca, 19. Yucatán.

The protected area network of Mexico (Figure 4) and biogeographic provinces (Figure 5) show an uneven distribution of climatic suitability. Observing spatial changes demonstrates that protected areas and provinces have a decrease in low suitability zones and an increase in high suitability zones in the future scenario, respectively. In the case of protected natural areas, the possible decrease that will occur in the future in protected areas such as the Sierra Gorda *s.l.* (Sierra Gorda and Sierra Gorda de Guanajuato), Los Tuxtlas, and Tehuacán-Cuicatlán Valley is notable. Some exceptions to this trend are the Flora and Fauna Protection Area Cuenca Alimentadora del Distrito Nacional de Riego 043, Estado de Nayarit, as well as the biosphere reserves of the Sierra de Manantlán (2), Monarch Butterfly (4) and El Triunfo (7). The models estimate in these areas that diversity could be maintained or increased in the long term, although this is uncertain. When modeling the climate change scenario on the map of biogeographic provinces, the results are similar. The current scenario shows that the greatest diversity is found along the Pacific coast, and in the Balsas Depression, Sierra Madre Occidental, Sierra Madre del Sur, Oaxaca, Altos de Chiapas, Soconusco, and Trans-Mexican Volcanic Belt. It is estimated that in the future, there will be a decrease in the Sierra Madre Occidental, Sierra Madre Oriental, and Altos de Chiapas. On the other hand, the difference between the two models indicates that there will be a small increase in the Trans-Mexican Volcanic Belt, Northern Altiplano (Chihuahuan desert) and Cape provinces, thereby maintaining the trend observed in the other models: a decrease in the sites that currently host the highest species richness, as well as an increase or no change in areas were the actual daisy tree diversity is considerable, such as the Trans-Mexican Volcanic Belt, Balsas Depression, Sierra Madre del Sur, Pacific coast, and Soconusco.

### 2.7. Mexican Daisy Tree Conservation

In the recently updated version of the Mexican decree of endangered species NOM-059-SEMARNAT-2010 [30], only 11 species of Asteraceae are included, of which none correspond to trees, despite the fact that some of them are only known from a few collections, or from the type collection only, and are distributed in areas with strong anthropogenic pressures derived from the change in land use, such as in the Uxpanapa-Chimalapas area in the states of Veracruz and Oaxaca. No Mexican species of Asteraceae are included in the updated appendices of the Convention of International Trade in Endangered Species of Wild Fauna and Flora [31], where only one species of Asteraceae is found—*Aucklandia costus* Falc. (cited as *Saussurea costus* (Falc.) Lipsch.)—due to its use in traditional Chinese medicine [32].

With respect to our ongoing assessments of the conservation status of the Mexican Asteraceae trees for the IUCN Red List, less than 10 will be categorized as critically endangered (CR), an estimated 15 to 20 as endangered (EN), about 20 to 25 as vulnerable, and the remainder as species of least concern (LC). The assessments with final conservation statuses will be published on the IUCN Red List later this year.

## 3. Discussion

### 3.1. Daisy Tree Diversity in Mexico and Comparison with Other Diverse Areas

The study by Beech et al. [25] reported 3364 tree species for Mexico, positioning this country in the top 10 of the most tree species-rich countries, and due to our efforts in listing additional tree species since then, this has been increased to 3522 species [33]. Similarly, for native Mexican Asteraceae trees, in contrast to the previous studies and reports, we report a much higher number of arborescent Asteraceae taxa. Asteraceae is a main component of vegetation and bioregions along the Americas, with Mexico standing out as the most species-rich country for this family at a global level [1,2].

With respect to the tree species richness of Asteraceae in megadiverse countries and areas, Mexico and Central America rank second in the number of genera and species with 45 and 149, respectively. The first corresponds to Colombia as it is home to 169 species [7], followed by Brazil with 38 genera [34], and Ecuador, with 14 genera and 55 species, all of them in some risk category according to the IUCN Red List criteria [8]. Considering regional scales, the Amazonian (an area that includes the territory of nine South American countries: Bolivia, Brazil, Colombia, Ecuador, French Guyana, Guyana, Peru, Suriname, and Venezuela) is an area of high diversity with 37 genera and 107 species [35]. With respect to the number of Asteraceae trees registered in the flora of North America [4], eight genera and 10 species were found, of which five are mainly shared with the northern part of Mexico; hence, the high diversity of daisy trees in the country stands out. A similar situation occurs with the flora of China [6]; in this case, the numbers are even more contrasting, since in that area only five genera and nine species of native trees are registered. In addition, in China there are also two genera and three cultivated species that are native to Mexico or Central America and that have become naturalized in Chinese territory. These are the only daisy tree species shared between Mexico, Central America, and that region in Asia.

Tree-like Asteraceae are generally not prominently visible in the forests where they occur, as they grow in the understory or in open places, whereas they can reach up to 20 m in cloud forest, but they do not form a prominent part of the forest structure. In contrast, in scrubland vegetation and semi-evergreen low forest, they may be dominant, and an important part of the structure of the forest. It has been documented that Mexican coniferous forests show a relationship between forest structure and tree diversity [36]. In the particular case of Asteraceae, the highest number of arborescent species is found in pine forests (71.8%), while *Abies* forests concentrate just over 11% of the 149 species present in the country. Even considering that one and the same species can be found in various vegetation types, the percentages for pine and *Abies* forests are considerable.

### 3.2. Uses of Mexican Daisy Trees

A considerable amount of ruderal or malezoid Asteraceae are nectariferous and therefore are particularly important for honey-producing bees [37,38,39,40], or other pollinators, which are also attracted in addition to nectar, by the yellow colour of the flowers of many species [41]; hence, they do not depend on a single vector that carries out cross-pollination. The nectar produced by the Asteraceae is rich in glucose, fructose and sucrose [42], which encourages various groups of insects, including Hymenoptera, Diptera, Lepidoptera, and Coleoptera, to obtain food and assist in the pollination of these species [42,43,44]. Even several groups within Mutisieae are hummingbird-pollinated [45,46]. The family is of high economic importance as a honey supplier in several regions in Mexico. The worldwide known migratory phenomenon of the monarch butterfly occurs every year during the fall when millions of butterflies travel from the south of Canada and the north of the United States of America to Mexico to spend the winter season in the Monarch Butterfly Biosphere Reserve, located on the limits of Michoacán and the State of Mexico [47]. The presence of 103 Asteraceae species has been documented in its core zone [48]. From the illustrated flora of the Reserve [49], it can be observed that the butterflies feed on practically all Asteraceae that grow in the area.

Asteraceae are also an important source of food for honey-producing bees in Mexico, both European and native [37,38,40]. Among the species most used by these insects, there is a significant percentage of those that have been classified as “weeds” [40]. Indirectly, these plants are a source of income for beekeepers around the world. Mexico is among the top 10 honey producers worldwide, ranking fourth in exports of this product. In 2019 alone, 61.9 million tons of honey were produced, thus achieving an increase of just over six percent compared to the previous five-year period [50]. Eight states account for 70% of the national production, with Yucatán, Campeche, Jalisco, and Chiapas as the main producers [51].

Asteraceae are used in traditional medicine to treat various conditions such as the treatment of stomach and respiratory diseases, since around 6000 species contain sesquiterpene lactones, chemical compounds with antimicrobial, antiprotozoal, anti-inflammatory and cytotoxic properties [44,52,53]. Others are used as food, whether they are cultivated to obtain leaves or meristems, roots, tubers, heads, seeds to produce oil, natural dyes, bio-insecticides, or as ornamental or florist plants [52]. Although only eight Mexican daisy tree species are used for ornamental purposes, it is important to highlight the potential that other species of the family could have, since, considering their importance as species that produce nectar and pollen, they would be helpful in reducing the loss of bees and other pollinator groups.

The dahlia deserves a special mention as it has been the national flower of Mexico since 1963 [54], since the country has the largest number of wild and endemic species, with 38 and 35, respectively. These are a source of germplasm for the more than 50,000 varieties grown around the world [55]. In addition to the dahlia, there are other wild species with ornamental potential due to their visible inflorescences, among which the following stand out: *Montanoa bipinnatifida* and *Bartlettina sordida*. The first is highly appreciated in Mexico [56,57], Spain [58], Australia [59], and New Zealand [60], and the second in Spain [61]. Although there are no published data, some shrub or tree species of Asteraceae are used as living fences, in seasonal crops such as corn or beans, or in vegetables or gardens, among which the following stand out: *Barkleyanthus salicifolius*, *Baccharis heterophylla*, *Baccharis salicifolia*, *Montanoa tomentosa*, *M. leucantha*, and *M. grandiflora*. Asteraceae species associated with corn (milpa) are mainly *Tithonia tubiformis*, *Cosmos bipinnatus*, *C. sulphureus*, *Bidens odorata*, *B. pilosa*, *Melampodium perfoliatum*, *Simsia amplexicaulis*, and *Viguiera dentata*, weedy plants that farmers allow to grow alongside the milpa to serve as crop protection, as the loss of the harvest is lessened in the case of a grasshopper or locust plague [29] (pers. obs.).

### 3.3. Distribution, Including Characterization of Climatic Suitability

The largest quantity of daisy trees is found in the west, center, and south of the country, particularly where the important mountain ranges of the Sierra Madre Occidental, Sierra Madre del Sur, and Trans-Mexican Volcanic Belt converge. Based on the results obtained from the climate suitability model in the future (2080 to 2100), populations will tend to decrease in the sites that are currently particularly rich in tree-like Asteraceae species, e.g., Chiapas, Sierra Norte de Oaxaca, as well as the northern portion of the Sierra Madre Occidental, in the territory occupied by the states of Durango and Sinaloa. There will also be a considerable decrease in the number of species in the Sierra Madre del Sur, particularly in Guerrero, a state that currently ranks fourth in species richness at a national level [3]. If these predictions materialize, several populations of species that are currently found in sites considered Pleistocene refugia [62,63,64] would be lost. This may be due to the fact that their ecosystems would not withstand a scenario of abrupt climate change, such as the one that is estimated to occur in the next 80 years [65]. In this way, the states with the greatest diversity of Asteraceae—Oaxaca, Jalisco, Durango, Guerrero, and Michoacán [3]—would lose a significant number of species and endemism (Figure 2, Figure 3 and Figure 4).

### 3.4. Conservation

The occurrence points of the known records, as well as the potential distribution models of Asteraceae trees compared with the areas occupied by the main natural protected areas present in the country, show a tendency to reduce their presence in some areas that are currently particularly rich in species; however, in other zones they will remain or increase; some of these correspond to protected natural areas.

Mexico has 182 protected natural areas distributed in maritime and continental territory. Of these, 67 correspond to National Parks, 44 are Biosphere Reserves, 40 Flora and Fauna Protection Areas, 18 Sanctuaries, eight Natural Resources Protection Areas, and five Natural Monuments [66]. Those that are located in continental territory are equivalent to 10.88% of the country’s land surface [66]. One of the terrestrial protected areas with the largest territorial extension is the Flora and Fauna Protection Area Cuenca Alimentadora del Distrito Nacional de Riego 043, Estado de Nayarit, located in the west of the country, comprising part of the territory of the states of Aguascalientes, Jalisco, Durango, Nayarit, and Zacatecas [66]. It is home to around 11 types of vegetation, more than 2000 species of vascular plants and at least two endemic daisy tree species [67,68]. The territorial extension and biological diversity of this protected area is considerable. In the particular case of Asteraceae trees, the climatic suitability models estimate that the diversity of species will remain in the western part of the country, a situation similar to what could occur in the Sierra de Manantlán, one of the most important biosphere reserves in the western region with an area of 139,577.12 ha [66]. The latter was recognized as a biosphere reserve for the biological diversity that it houses in its territory, which includes vegetation of dry, temperate, and humid environments, in addition to being the main water source for more than 430,000 inhabitants of southern Jalisco and northern Colima [69].

The biosphere reserves are distributed throughout the country; 70% have territorial extensions of more than 100 ha or a high species diversity [66]. In the center of the country, the Sierra Gorda stands out with an extension of 383,567.45 ha, located on the limits of Querétaro, Guanajuato, San Luis Potosí and Hidalgo, and Sierra Gorda de Guanajuato (236,882.76 ha) [66], which together occupy the seventh place in size of all the protected natural areas of Mexico [70]. In its territory, there are dry shrublands and temperate forests, which host a great diversity of plants, many of them endemic to the Sierra Madre Oriental [70]. This region has three hydrological sub-basins and a dam declared a Ramsar site, as it is a wetland of global importance [70]. The climate suitability models estimate a slight decrease in the number of tree species of Asteraceae (Figure 4). Among those that would be affected are *Baccharis heterophylla*, *Barkleyanthus salicifolius*, *Critonia morifolia*, *Koanophyllon albicaulis*, and *Nahuatlea hypoleuca*, species that fortunately are not restricted to Mexico, since the first four also occur in Central America and the fifth in the south of the United States of America.

One of the most important protected areas in central Mexico is the Monarch Butterfly Biosphere Reserve with an area of 56,259.05 ha [66]. In its territory, there are pine forests, *Abies* forests, and oak forests that contribute in an important way to the carbon capture from industrial areas and favour the recharge of aquifers that provide water to the metropolitan area of Mexico City, as well as various areas of the states of Michoacán and Mexico [47]. Our models estimate that the daisy tree diversity in this region will be maintained in the next century, if this also happens with the ecosystems where they are found, as well as with the environmental services that they provide to this area of the country.

The Tehuacán-Cuicatlán Valley Biosphere Reserve, with a surface area of 490,186.87 ha, is the largest of all those found in arid and semi-arid zones [66]. In this case, the climatic suitability models estimate a possible decline in species (Figure 3, Figure 4 and Figure 5). Although the number of trees that are currently in its territory is minimal, several species dominate and give structure to the vegetation of some areas, among them: *Baccharis heterophylla*, *Montanoa leucantha*, *M. tomentosa*, *Nahuatlea hypoleuca*, *N. smithii*, *Parthenium tomentosum*, *Pittocaulon praecox*, *P. velatum*, *Roldana eriophylla*, and *R. oaxacana*. Of these, *Nahuatlea smithii* and *Roldana eriophylla* are practically endemic to this region and could be at risk if the scenario predicted by the models takes place.

In the case of tropical ecosystems and particularly rainforests, the main biosphere reserves are: Montes Azules (331,200 ha) and El Triunfo (119,177.29 ha) in Chiapas, and Los Tuxtlas (155,122.47 ha), in Veracruz [66]. Interestingly, the three are in territory belonging to Pleistocene refugia; Montes Azules corresponds to the refugium called Lacandonia and El Triunfo to Soconusco [62]. Both are considered primary Pleistocene refugia; i.e., these zones maintained constant temperature and precipitation conditions during the dry and cold periods that occurred during this period, which allowed them to safeguard common species in humid tropical forests [62]. The climate suitability models estimate different situations in the event of abrupt climatic events, since a probable reduction would occur in Montes Azules, while in El Triunfo, the Asteraceae tree populations would remain. This may be due to the climatic conditions that currently prevail in each of these regions, since in Montes Azules the current oscillation of temperature and precipitation is lower (average annual temperature between 22 and 24 °C; average annual precipitation 2000 to 3000 mm [71]), compared to the values recorded in El Triunfo (mean annual temperature of 18 to 22 °C, with a mean annual rainfall of 1000 to 3000 mm [72]). However, both sites have invaluable biological potential and value. Montes Azules protects 20% of the plant species present in Mexico; recent calculations indicate that one hectare of this reserve protects about 160 tree species and about 700 vascular plants [73]. In El Triunfo, there are important extensions of mountain cloud forest, considered one of the ecosystems that hosts the greatest diversity of trees in North and Central America [74]. The Los Tuxtlas reserve is located in an area recognized as a secondary Pleistocene refugium, because it corresponds to an area that only managed to preserve itself from the drastic drop in temperature or precipitation, during the alternation of cold and dry periods of the Pleistocene [62].

Los Tuxtlas homes coniferous forest, oak forest, mountain cloud forest, high evergreen forest, and mangroves, where around 3000 plant species have been documented, and it is also one of the five areas with the highest amount of tree endemism in Mexico [75]. For this site, the climatic suitability models estimate a decrease in the number of daisy trees. Considering the importance of Los Tuxtlas as an area of endemism for tree species, it would be interesting to explore what happens with other angiosperm groups. In this area, the average annual temperature ranges between 22 and 26 °C and rainfall varies from 1500 to 4500 mm [76]. Although the temperature is relatively constant, the precipitation has a considerable range of variation, which would support the proposal that it was a secondary Pleistocene refugium [62].

Apparently, the aforementioned biosphere reserves will allow that, given a scenario such as that estimated by our models, the probable reduction of the populations does not become catastrophic as in other areas of the country, which are particularly rich in diversity and Asteraceae endemisms, as occurs with the Tehuacán-Cuicatlán Valley.

Anthropocene refugia correspond to territories that meet the following qualities: being ecologically suitable areas to house the diversity units analyzed and having relatively low levels of observed and predicted anthropogenic pressure to allow their long-term persistence in this area, i.e., through several generations [77]. The main difference between Pleistocene and Anthropocene refugia is that the former are sites where organisms resisted and responded to glacial and interglacial oscillations of the late Quaternary, having the possibility of expanding their distribution once environmental stress conditions decreased [78]. In contrast, in order to characterize probable refugia from the Anthropocene, climate change derived from anthropogenic pressures is taken into account [77]. Therefore, the identification of Anthropocene refugia is useful to categorize, plan, and decide where to establish conservation areas for the group of interest [77].

In the case of daisy trees, the models allow the identification of some areas that meet sufficient characteristics to be considered Anthropocene refugia and, therefore, to be maintained or proposed as conservation areas, although some of them are already cataloged like this. This is the case of the western region of Jalisco where the Flora and Fauna Protection Zone Cuenca Alimentadora del Distrito Nacional de Riego 043, Estado de Nayarit and the Biosphere Reserve Sierra de Manantlán are located. Moreover, the Biosphere Reserves of the Monarch Butterfly, on the limits of Michoacán and the State of Mexico, and of the Tacaná Volcano and El Triunfo in Chiapas are also already existing conservation areas; the latter has previously been proposed as a Pleistocene refugium [62]. Based on the results obtained, other areas that could function as Anthropocene refugia correspond to the northern portion of Michoacán and southern part of the State of Mexico, which together with western Jalisco form part of the Trans-Mexican Volcanic Belt, an area that currently contains a high richness of Asteraceae trees, which, according to our models, is estimated to remain or increase (Figure 3, Figure 4 and Figure 5). The mountainous regions of Guerrero and Oaxaca, corresponding to the Sierra Madre del Sur (Figure 3), also seem to meet the characteristics of Anthropocene refugia. Although there are currently no natural protected areas decreed in these areas, despite being sites with high biological diversity and a large number of endemisms, it is a fact that their geographical location and the difficulty of accessing them has kept them safe from human damage. Special mention should be made of the Sierra Norte de Oaxaca, an area particularly rich in diversity and endemism of Asteraceae [79], in which the models indicate that there are also adequate conditions to serve as an Anthropocene refugium, which is confirmed by the fact that it has also been considered as a secondary Pleistocene refugium [62]. However, unlike the mountainous region of central Oaxaca, the Sierra Norte has sufficient infrastructure to access its territory and forest management of the coniferous forests, although the other ecosystems remain almost intact. This shows that, despite the fact that this area is not recognized as a protected area at the federal level, the community forest management that the inhabitants of the region have carried out has been adequate and successful, since in addition to generating jobs and resources for the inhabitants of the region, the forest area has increased in the last four decades [80,81,82,83].

Based on the aforementioned, the sites identified as priority areas to conserve the diversity and endemism of daisy trees are the following: Trans-Mexican Volcanic Belt (including Protected Areas of western of Jalisco), Sierra Madre del Sur, Sierra Norte de Oaxaca, El Triunfo and Tacaná Volcano. All these regions have previously been identified as diversity hotspots of other groups of plants [84,85] and animals [86].

As a consequence, whether the efforts and proposals to conserve nature are federal or local, everything seems to indicate that the establishment, maintenance and conservation of protected natural areas that currently exist in Mexico have been adequate. However, the ideal would be to keep them intact in the long term or, as far as possible, to extend their territory in order to safeguard a greater number of species, both Asteraceae and other families of angiosperms.

In conclusion, relatively few Mexican daisy tree species are currently seriously threatened by climate change or other factors, as most species are widely distributed. Direct exploitation for human use is also not generally a threatening factor. Mexico ranks first at the global level with respect to daisy diversity and second with respect to daisy tree diversity. As mentioned above, Asteraceae are ecologically successful, and the same goes for tree-like representatives of this family. However, it will be important to include those endemic species whose IUCN Red List assessment indicates that they are endangered or critically endangered in the NOM-059-SEMARNAT-2010, as several of these, e.g., *Ageratina chimalapana*, *Lepidonia wendtiana*, *Mixtecalia teitaensis*, *Montanoa revealii*, and *Verbesina sousae*, occur in areas subject to anthropogenic pressures that puts their survival at risk, either due to changes in land use, excessive or unplanned tourism, and the extraction of stone material.

## 4. Materials and Methods

### 4.1. Study Area

Mexico is located in North America, between the extreme coordinates 32°43′06′′ and 19°32′25′′ latitude N and 114°43′22′′and 84°38′30′′ longitude W. It limits to the north with the United States of America and to the south with Belize and Guatemala. It has a territorial extension of 1,960,189 km^2^; which positions it in sixth place among the American countries and as 14th in the world. Nearly half of the country is located below the Tropic of Cancer, which favours the presence of temperate and cold climates in the north, as well as temperate and warm climates in the south. In addition, the geographical position of the country, the presence of large mountain ranges such as the Sierra Madre Oriental to the east, the Sierra Madre Occidental to the west and the Trans-Mexican Volcanic Belt that crosses the territory from east to west in the central-southern area of the country, together with geological, edaphic, and microclimatic variations, favour the existence of a great diversity of vegetation types and therefore a high biodiversity [87]. Mexico shares floristic diversity with neighbouring countries; in the northern part it has affinities with some regions of the United States of America, in the southern part with Central America, and to a lesser extent with South America. The species analyzed in this work are those that are endemic or near-endemic to Mexico, i.e., those shared with the south of the United States of America north of Mexico, and those shared with Central America south of the country.

For the purpose of this study, we only focus on the occurrence, distribution pattern uses, and conservation of the daisy tree species within the Mexico territory.

### 4.2. Compilation of Taxonomic List and Species Information (Use and Habitat)

From a bibliographic review and the consultation of herbarium specimens available online, a list of tree species of Asteraceae was made (Appendix A), considering in this category those that have been described as trees, arborescent or small trees and even some that have been registered as shrubs, but that sometimes also develop a tree habit according to the definition we used. This list was generated based on the revision of regional floras, such as: Flora Novogaliciana [56], Flora of Chiapas [14], Flora Mesoamericana [88], treatments of the Flora del Valle de Tehuacán-Cuicatlán [89,90,91], Flora del Bajío y de Regiones Adyacentes [57]; taxonomic reviews at tribe level (Eupatorieae [13]), genus level [15,16,17,18,19,20,21,92,93], and section level [22,23]. Moreover, online available collections were also consulted: the National Herbarium of Mexico (MEXU) [29], United States National Herbarium (US) [94], herbarium of the Missouri Botanical Garden (MO) [95], and several herbaria of northern Mexico and Arizona whose collections are available via the portal SEinet Arizona-New Mexico Chapter [96].

In addition to the compilation of Mexican species, we looked up the number of daisy tree species in at least five other megadiverse countries: Brazil [5], China [6], Colombia [7], Ecuador [8], and the United States of America [4].

The most recent classification of Asteraceae for subfamilies and tribes is used in Appendix A [28]. The names of some genera and species are based on specialist reviews and criteria, for example, *Ageratina* [13,97], *Critoniopsis* [98], and *Pachythamnus* [99].

### 4.3. Compilation of Species Ocurrence Geographical Data

The geographical coordinates were obtained from GBIF [100] via GeoCAT [101], carrying out an exhaustive curation of the information, which consisted mainly of eliminating records of human observations without support by vouchers or photos, as well as those that lacked information regarding the collector or herbarium where the voucher is located, or that only presented decimal coordinates. Moreover, duplicate records in the same locality were also deleted. Records that did not correspond to the known distribution of the species were also eliminated, based on expert knowledge, either due to possible misidentifications or because they correspond to specimens grown outside the natural distribution area of a certain species, or invasive groups in areas other than their natural distribution.

When the records were minimal or there was no information available in GBIF [100], this was complemented with data obtained from the labels of herbarium specimens available online through the digital platforms of the National Herbarium of Mexico (MEXU) [29], the National Herbarium of the United States of America (US) [94] and several herbaria of Northern Mexico and Arizona, USA [96]. The localities that lacked geographical coordinates were georeferenced using Google Earth Pro [102]. To locate some little-known localities, the Historical Archive of geostatistical localities [103], Mapcarta [104], and Pueblos de México [105] were used. The data for each species were generated in Excel tables in comma delimited text format (.csv).

### 4.4. Spatial Analyses

A species distribution modelling (SDM) approach was used to characterise the spatial patterns of tree Asteraceae species in Mexico. SDM allows identifying the geographic areas with the highest climatic suitability in the current period and projects this suitability in future scenarios. The modelling approach was maximum entropy using Maxent software [106]. To model climate adequacy, the compiled occurrence database is used together with a set of explanatory climate variables. Only species with more than 50 unique occurrences were selected for modelling, to ensure good performance [107]. In this study, the climate variables were obtained from the Worldclim 2 database [108] for the current period (1970 to 2000). The variables were selected from the set of 19 bioclimatic variables available in Worldclim at a spatial resolution of 1 km, which were analysed for their degree of correlation in the Americas’ total extent of occurrence. Correlation values between variables higher than 0.7 were excluded, obtaining a set of six variables with low correlation. The variables were temperature seasonality (BIO4), minimum temperature of coldest month (BIO6), temperature annual range (BIO7), annual precipitation (BIO12), precipitation seasonality (BIO15), and precipitation of coldest quarter (BIO19). Each species’ projection was carried out by maintaining the default Maxent regularisation parameters (auto features) and avoiding extrapolation and clamping options. Records less than 1 km apart per species were excluded to avoid spatial autocorrelation. Occurrences were divided into a training set (70% of the total) and a test set (30%). Maxent probability models were projected over the entire distribution of the species occurrences and then were restricted to the continental area of Mexico by cropping the total raster extent area. This step was done to avoid the loss of potential climatic space combinations where species are present outside Mexico and improving final model accuracy [109]. Models were transformed into a binary format, using a threshold of maximum training sensitivity plus specificity [110]. The binary models per species were summed to obtain the current climate suitability pattern. Future projections were obtained using the global circulation model MIROC6 [111], which has been assessed to represent the average conditions of different climatic factors on a global scale [112]. From this global circulation model, the most extreme scenario SSP585 was selected for the future period 2081 to 2100. The same transformation procedure to binary and summation by species was repeated to obtain the future pattern of climate suitability. Three spatial analyses were performed with the current and future climate suitability models: the calculation of the difference between the future and current patterns, the extraction of the climate suitability in Mexico’s protected areas, and finally the extraction for the biogeographic provinces of Mexico [113]. All spatial analyses were performed with ESRI Arcgis software (version 10.8).

### 4.5. IUCN Red List Assessments

Red List categories were applied according to the IUCN red list criteria [114] and all relevant information was completed in the IUCN SIS database for pending publication on the publicly available IUCN Red List. Data on species occurrence, uses and habitat are those that were obtained for the abovementioned analyses.

## Figures and Tables

**Table 1:** Number of Mexican Asteraceae tree species grouped by subfamilies and tribes based on the most recent classification by Susanna et al. [28]. The percentage represents the number of species of each tribe with respect to the 149 that represent the family in Mexico.

Subfamily	Tribe	Species	Percentage
Gochnatioideae	Gochnatieae	3	2.01%
Vernonioideae	Liabeae	1	0.67%
	Vernonieae	16	10.73%
	Senecioneae	20	13.42%
	Astereae	4	2.68%
	Inuleae	1	0.67%
	Neurolaeneae	1	0.67%
Asteroideae	Millerieae	3	2.01%
	Coreopsideae	2	1.34%
	Bahieae	1	0.67%
	Heliantheae	55	36.9%
	Eupatorieae	42	28.18%

**Table 2:** Use of Mexican arborescent Asteraceae species.

Use	Category	Species
Medicinal	Oral diseases	3
	Heart diseases	1
	Stomach diseases	11
	Skin diseases	10
	Gynecological diseases	2
	Anticonceptive	2
	Anti-inflammatory	12
	Antiseptic	5
	Diuretic	2
	Fever reducer	5
	Reuma	4
	Vertigo	1
	Various	8
Nectariferous	Honeybees	17
	Butterflies	3
	Hummingbirds	1
Ornamental	Live fence	2
	Cut flower	2
	Decoration	8
Others	Artesanal	2
	Ceremonial	3
	Fuel	8
	Construction	3
	Forage	6
	Insecticide	1
	Ritual	8
	Shade for coffee	3

## Data Availability

All data used in this study were obtained from databases and publicly available literature sources, all of them were cited in the references.

## Appendix A

**Table A1:** List of Mexican endemic and near-endemic arborescent Asteraceae species.

Subfamily	Tribe	Species
Gochnatioideae	Gochnatieae	*Nahuatlea arborescens* (Brandegee) V.A. Funk
	Gochnatieae	*Nahuatlea hypoleuca* (DC.) V.A. Funk
	Gochnatieae	*Nahuatlea smithii* (B.L. Rob. & Greenm.) V.A. Funk
Vernonioideae	Liabeae	*Sinclairia glabra* (Hemsl.) Rydb.
	Vernonieae	*Critoniopsis baadii* (McVaugh) H. Rob.
	Vernonieae	*Critoniopsis heydeana* (J.M. Coult.) H. Rob.
	Vernonieae	*Critoniopsis leiocarpa* (DC.) H. Rob.
	Vernonieae	*Critoniopsis macvaughii* (S.B. Jones) H. Rob.
	Vernonieae	*Critoniopsis obtusa* (Gleason) H. Rob.
	Vernonieae	*Critoniopsis salicifolia* (DC.) H. Rob.
	Vernonieae	*Critoniopsis shannonii* (J.M. Coult.) H. Rob.
	Vernonieae	*Critoniopsis tomentosa* (Lex.) H. Rob.
	Vernonieae	*Critoniopsis triflosculosa* (Kunth) H. Rob.
	Vernonieae	*Critoniopsis uniflora* (Sch. Bip.) H. Rob.
	Vernonieae	*Critoniopsis villaregalis* (Carvajal) H. Rob.
	Vernonieae	*Lepidaploa polypleura* (S.F. Blake) H. Rob.
	Vernonieae	*Lepidonia salvinae* (Hemsl.) H. Rob. & V.A. Funk
	Vernonieae	*Lepidonia wendtiana* (B.L. Turner) Redonda-Mart. & Villaseñor
	Vernonieae	*Vernonanthura cordata* (Kunth) H. Rob.
	Vernonieae	*Vernonanthura patens* (Kunth) H. Rob.
Asteroideae	Astereae	*Baccharis glandulifera* G.L. Nesom
	Astereae	*Baccharis heterophylla* Kunth
	Astereae	*Baccharis lancifolia* Less.
	Astereae	*Baccharis salicifolia* (Ruiz & Pav.) Pers. subsp. *monoica* (G.L. Nesom) Joch. Müll.
	Bahieae	*Peucephyllum schottii* A.Gray
	Coreopsideae	*Dahlia imperialis* Roezl ex Ortgies
	Coreopsideae	*Electranthera mutica* (DC.) Mesfin, D.J.Crawford & Pruski
	Eupatorieae	*Ageratina areolaris* (DC.) Gage ex B.L.Turner
	Eupatorieae	*Ageratina cerifera* (McVaugh) R.M. King & H. Rob.
	Eupatorieae	*Ageratina chiapensis* (B.L. Rob.) R.M. King & H. Rob.
	Eupatorieae	*Ageratina chimalapana* B.L. Turner
	Eupatorieae	*Ageratina cylindrica* (McVaugh) R.M. King & H. Rob.
	Eupatorieae	*Ageratina glabrata* (Kunth) R.M. King & H. Rob.
	Eupatorieae	*Ageratina grandifolia* (Regel) R.M. King & H. Rob.
	Eupatorieae	*Ageratina havanensis* (Kunth) R.M. King & H. Rob.
	Eupatorieae	*Ageratina ligustrina* (DC.) R.M. King & H. Rob.
	Eupatorieae	*Ageratina mairetiana* (DC.) R.M.King & H.Rob.
	Eupatorieae	*Ageratina vernalis* (Vatke & Kurtz) R.M. King & H. Rob.
Asteroideae	Eupatorieae	*Amolinia heydeana* (B.L.Rob.) R.M.King & H.Rob.
	Eupatorieae	*Bartlettina luxii* (B.L. Rob.) R.M. King & H. Rob.
	Eupatorieae	*Bartlettina pansamalensis* (B.L. Rob.) R.M. King & H. Rob.
	Eupatorieae	*Bartlettina pinabetensis* (B.L. Rob.) R.M. King & H. Rob.
	Eupatorieae	*Bartlettina platyphylla* (B.L. Rob.) R.M. King & H. Rob.
	Eupatorieae	*Bartlettina prionophylla* (B.L. Rob.) R.M. King & H. Rob.
	Eupatorieae	*Bartlettina sordida* (Less.) R.M. King & H. Rob.
	Eupatorieae	*Bartlettina tuerckheimii* (Klatt) R.M. King & H. Rob.
	Eupatorieae	*Bartlettina williamsii* R.M. King & H. Rob.
	Eupatorieae	*Chromolaena collina* (DC.) R.M. King & H. Rob.
	Eupatorieae	*Chromolaena glaberrima* (DC.) R.M.King & H.Rob.
	Eupatorieae	*Critonia breedlovei* R.M. King & H. Rob.
	Eupatorieae	*Critonia conzatti* (Greenm.) R.M. King & H. Rob.
	Eupatorieae	*Critonia daleoides* DC.
	Eupatorieae	*Critonia hebebotrya* DC.
	Eupatorieae	*Critonia hospitalis* (B.L. Rob.) R.M. King & H. Rob.
	Eupatorieae	*Critonia iltisii* R.M. King & H. Rob.
	Eupatorieae	*Critonia morifolia* (Mill.) R.M. King & H. Rob.
	Eupatorieae	*Critonia paneroi* B.L.Turner
	Eupatorieae	*Critonia quadrangularis* (DC.) R.M. King & H. Rob.
	Eupatorieae	*Critonia sexangularis* (Klatt) R.M. King & H. Rob.
	Eupatorieae	*Critonia tuxtlae* R.M. King & H. Rob.
	Eupatorieae	*Critoniadelphus microdon* (B.L. Rob.) R.M. King & H. Rob.
	Eupatorieae	*Critoniadelphus nubigenus* (Benth.) R.M. King & H. Rob.
	Eupatorieae	*Koanophyllon albicaule* (Sch. Bip. ex Klatt) R.M. King & H. Rob.
	Eupatorieae	*Koanophyllon galeottii* (B.L. Rob.) R.M. King & H. Rob.
	Eupatorieae	*Koanophyllon palmeri* (A. Gray) R.M. King & H. Rob.
	Eupatorieae	*Koanophyllon pittieri* (Klatt) R.M. King & H. Rob.
	Eupatorieae	*Koanophyllon revealii* B.L. Turner
	Eupatorieae	*Kyrsteniopsis nelsonii* (B.L. Rob.) R.M. King & H. Rob.
	Eupatorieae	*Pachythamnus crassirameus* (B.L. Rob.) R.M. King & H.Rob.
	Heliantheae	*Clibadium arboreum* Donn. Sm.
	Heliantheae	*Clibadium leiocarpum* Steetz in Seemann
	Heliantheae	*Clibadium surinamense* L.
	Heliantheae	*Dendroviguiera puruana* (Paray) E.E. Schill. & Panero
	Heliantheae	*Dendroviguiera quinqueradiata* (Cav.) E.E. Schill. & Panero
	Heliantheae	*Dendroviguiera sphaerocephala* (DC.) E.E. Schill. & Panero
	Heliantheae	*Dendroviguiera splendens* (Panero & E.E. Schill.) E.E. Schill. & Panero
	Heliantheae	*Lagascea palmeri* (B.L. Rob.) B.L. Rob.
	Heliantheae	*Lasianthaea fruticosa* (L.) K.M. Becker
	Heliantheae	*Montanoa andersonii* McVaugh
Asteroideae	Heliantheae	*Montanoa bipinnatifida* (Kunth) K. Koch
	Heliantheae	*Montanoa frutescens* (Mairet ex DC.) Hemsl.
	Heliantheae	*Montanoa grandiflora* Alamán ex DC.
	Heliantheae	*Montanoa hexagona* B.L. Rob. & Greenm.
	Heliantheae	*Montanoa imbricata* V.A. Funk
	Heliantheae	*Montanoa karwinskii* DC.
	Heliantheae	*Montanoa leucantha* (Lag.) S.F. Blake
	Heliantheae	*Montanoa revealii* H. Rob.
	Heliantheae	*Montanoa speciosa* DC.
	Heliantheae	*Montanoa tomentosa* Cerv.
	Heliantheae	*Parthenium fruticosum* Less. ex Schltdl. & Cham.
	Heliantheae	*Parthenium schottii* Greenm. ex Millsp. & Chase
	Heliantheae	*Parthenium tomentosum* DC.
	Heliantheae	*Perymenium grande* Hemsl.
	Heliantheae	*Perymenium hintonii* McVaugh
	Heliantheae	*Podachaenium chiapanum* B.L. Turner & Panero
	Heliantheae	*Podachaenium eminens* (Lag.) Sch. Bip. ex Sch. Bip.
	Heliantheae	*Podachaenium standleyi* (Steyerm.) B.L.Turner & Panero
	Heliantheae	*Rensonia salvadorica* S.F. Blake
	Heliantheae	*Rojasianthe superba* Standl. & Steyerm.
	Heliantheae	*Squamopappus skutchii* (S.F. Blake) R.K. Jansen, N.A. Harriman & Urbatsch
	Heliantheae	*Tetrachyron orizabaensis* (Klatt) Wussow & Urbatsch
	Heliantheae	*Tithonia koelzii* McVaugh
	Heliantheae	*Tithonia longiradiata* (Bertol.) S.F. Blake
	Heliantheae	*Verbesina apleura* S.F. Blake
	Heliantheae	*Verbesina breedlovei* B.L. Turner
	Heliantheae	*Verbesina culminicola* McVaugh
	Heliantheae	*Verbesina fastigiata* B.L. Rob. & Greenm.
	Heliantheae	*Verbesina furfuracea* McVaugh
	Heliantheae	*Verbesina guatemalensis* B.L. Rob. & Greenm.
	Heliantheae	*Verbesina hypargyrea* B.L. Rob. & Greenm.
	Heliantheae	*Verbesina hypoglauca* Sch. Bip. ex Klatt
	Heliantheae	*Verbesina klattii* B.L. Rob. & Greenm.
	Heliantheae	*Verbesina lanata* B.L. Rob. & Greenm.
	Heliantheae	*Verbesina montanoifolia* B.L. Rob. & Greenm.
	Heliantheae	*Verbesina oncophora* B.L. Rob. & Seaton
	Heliantheae	*Verbesina oligantha* B.L. Rob.
	Heliantheae	*Verbesina ovatifolia* A. Gray
	Heliantheae	*Verbesina perymenioides* Sch. Bip. ex Klatt
	Heliantheae	*Verbesina platyptera* Sch. Bip. ex Klatt
	Heliantheae	*Verbesina sousae* J.J. Fay
Asteroideae	Heliantheae	*Verbesina sphaerocephala* A. Gray
	Heliantheae	*Verbesina turbacensis* Kunth
	Heliantheae	*Verbesina villaregalis* McVuagh
	Heliantheae	*Wamalchitamia aurantiaca* (Klatt) Strother
	Inuleae	*Pluchea sericea* (Nutt.) Coville
	Millerieae	*Desmanthodium perfoliatum* Benth.
	Millerieae	*Rumfordia floribunda* DC.
	Millerieae	*Schistocarpha longiligula* Rydb.
	Neurolaeneae	*Neurolaena macrophylla* Greenm.
	Senecioneae	*Barkleyanthus salicifolius* (Kunth) H. Rob. & Brettell
	Senecioneae	*Lepidospartum squamatum* (A. Gray) A. Gray
	Senecioneae	*Mixtecalia teitaensis* Redonda-Mart., García-Mend., & D. Sandoval
	Senecioneae	*Pittocaulon filare* (McVaugh) H. Rob. & Brettell
	Senecioneae	*Pittocaulon praecox* (Cav.) H. Rob. & Brettell
	Senecioneae	*Pittocaulon velatum* (Greenm.) H. Rob. & Brettell
	Senecioneae	*Roldana albonervia* (Greenm.) H. Rob. & Brettell
	Senecioneae	*Roldana angulifolia* (DC.) H. Rob. & Brettell
	Senecioneae	*Roldana barba-johannis* (DC.) H. Rob. & Brettell
	Senecioneae	*Roldana eriophylla* (Greenm.) H. Rob. & Brettell
	Senecioneae	*Roldana gentryi* H. Rob. & Brettell
	Senecioneae	*Roldana greenmanii* H. Rob. & Brettell
	Senecioneae	*Roldana neogibsonii* (B.L. Turner) B.L. Turner
	Senecioneae	*Roldana oaxacana* (Hemsl.) H.Rob. & Brettell
	Senecioneae	*Roldana schaffneri* (Sch. Bip. ex Klatt) H. Rob. & Brettell
	Senecioneae	*Telanthophora cobanensis* (J.M. Coult.) H. Rob. & Brettell
	Senecioneae	*Telanthophora grandifolia* (Less.) H.Rob. & Brettell
	Senecioneae	*Telanthophora jaliscana* H. Rob. & Brettell
	Senecioneae	*Telanthophora standleyi* (Greenm.) H. Rob. & Brettell
	Senecioneae	*Telanthophora uspantanensis* (J.M. Coult.) H. Rob. & Brettell

## Appendix B

**Table A2:** Species list and number of records used for the climatic suitability models. I. Records in whole distribution area, II. Records in Mexico, III. Current suitable area (km^2^), IV. Future suitable area (km^2^), V. Future range expansion in Mexico (km^2^), VI. Future range stability in Mexico (km^2^), VII. Future range contraction in Mexico (km^2^), VIII. Future range expansion in Mexico (%), IX. Future range stability in Mexico (%), X. Future range contraction in Mexico (%).

ID	Species	I	II	III	IV	V	VI	VII	VIII	IX	X
1	*Ageratina areolaris* (DC.) Gage ex B.L.Turner	552	538	439,361	258,128	37,770	167,455	184,977	9.0	39.8	43.9
2	*Ageratina chiapensis* (B.L. Rob.) R.M. King & H. Rob.	97	94	364,784	246,875	3687	10,875	237,846	1.0	3.0	65.2
3	*Ageratina grandifolia* (Regel) R.M. King & H. Rob.	68	68	412,400	139,148	1401	110,475	221,618	0.3	26.8	53.7
4	*Ageratina ligustrina* (DC.) R.M. King & H. Rob.	824	716	625,473	241,772	2746	191,062	309,808	0.4	30.5	49.5
5	*Ageratina mairetiana* (DC.) R.M.King & H.Rob.	577	567	387,878	198,528	12,833	146,160	165,799	3.3	37.7	42.7
6	*Ageratina vernalis* (Vatke & Kurtz) R.M. King & H. Rob.	106	102	557,923	183,660	3409	142,534	301,187	0.6	25.5	54.0
7	*Baccharis heterophylla* Kunth	502	499	650,100	444,872	45,738	307,814	211,270	7.0	47.3	32.5
8	*Baccharis lancifolia* Less.	71	58	372,699	119,928	4076	92,403	206,874	1.1	24.8	55.5
9	*Baccharis monoica* G.L. Nesom	146	55	336,151	128,384	3697	101,109	172,325	1.1	30.1	51.3
10	*Barkleyanthus salicifolius* (Kunth) H. Rob. & Brettell	416	411	871,464	563,600	41,211	402,173	283,498	4.7	46.1	32.5
11	*Bartlettina platyphylla* (B.L. Rob.) R.M. King & H. Rob.	77	37	279,330	118,849	3467	92,476	132,519	1.2	33.1	47.4
12	*Bartlettina sordida* (Less.) R.M. King & H. Rob.	179	176	311,664	64,849	258	51,960	199,004	0.1	16.7	63.9
13	*Bartlettina tuerckheimii* (Klatt) R.M. King & H. Rob.	146	132	420,782	100,855	95	81,591	258,139	0.0	19.4	61.3
14	*Chromolaena collina* (DC.) R.M. King & H. Rob.	742	608	862,983	918,322	189,840	531,576	156,402	22.0	61.6	18.1
15	*Chromolaena glaberrima* (DC.) R.M.King & H.Rob.	227	97	386,145	174,246	5085	136,090	176,815	1.3	35.2	45.8
16	*Clibadium arboreum* Donn. Sm.	509	443	312,076	118,778	9241	87,285	164,964	3.0	28.0	52.9
17	*Clibadium surinamense* L.	359	9	17,074	2561	1116	978	12,947	6.5	5.7	75.8
18	*Critonia daleoides* DC.	417	228	534,699	215,084	11,819	160,473	270,312	2.2	30.0	50.6
19	*Critonia hebebotrya* DC.	121	95	725,959	587,158	37,854	430,086	153,176	5.2	59.2	21.1
20	*Critonia hospitalis* (B.L. Rob.) R.M. King & H. Rob.	78	75	300,071	64,605	241	52,128	190,791	0.1	17.4	63.6
21	*Critonia morifolia* (Mill.) R.M. King & H. Rob.	802	301	532,910	281,050	36,494	188,731	241,060	6.8	35.4	45.2
22	*Critonia quadrangularis* (DC.) R.M. King & H. Rob.	147	112	823,247	1,190,328	288,440	648,289	5097	35.0	78.7	0.6
23	*Critonia sexangularis* (Klatt) R.M. King & H. Rob.	178	31	77,991	11,474	4005	5362	58,177	5.1	6.9	74.6
24	*Critoniadelphus nubigenus* (Benth.) R.M. King & H. Rob.	57	37	313,638	89,891	0	73,133	182,011	0.0	23.3	58.0
25	*Critoniopsis leiocarpa* (DC.) H. Rob.	315	221	311,807	103,670	1117	82,904	170,099	0.4	26.6	54.6
26	*Critoniopsis obtusa* (Gleason) H. Rob.	118	118	461,220	360,251	54,851	230,966	133,515	11.9	50.1	28.9
27	*Critoniopsis salicifolia* (DC.) H. Rob.	115	115	587,539	563,699	46,612	404,981	68,241	7.9	68.9	11.6
28	*Critoniopsis tomentosa* (Lex.) H. Rob.	225	225	426,071	293,206	27,519	206,722	136,001	6.5	48.5	31.9
29	*Critoniopsis uniflora* (Sch. Bip.) H. Rob.	170	267	686,446	717,520	121,094	443,992	103,775	17.6	64.7	15.1
30	*Dahlia imperialis* Roezl ex Ortgies	143	197	536,126	473,635	33,319	345,751	84,478	6.2	64.5	15.8
31	*Dendroviguiera quinqueradiata* (Cav.) E.E. Schill. & Panero	114	42	218,168	33,681	0	27,472	150,172	0.0	12.6	68.8
32	*Dendroviguiera sphaerocephala* (DC.) E.E. Schill. & Panero	110	114	182,080	173,000	36,608	101,219	44,420	20.1	55.6	24.4
33	*Desmanthodium perfoliatum* Benth.	94	110	328,888	198,100	5352	154,402	111,546	1.6	46.9	33.9
34	*Electranthera mutica* (DC.) Mesfin, D.J.Crawford & Pruski	730	94	264,289	105,968	4255	81,480	132,739	1.6	30.8	50.2
35	*Critoniopsis triflosculosa* (Kunth) H. Rob.	364	679	540,285	336,732	18,823	252,324	183,402	3.5	46.7	33.9
36	*Koanophyllon albicaule* (Sch. Bip. ex Klatt) R.M. King & H. Rob.	501	407	694,526	919,970	189,819	548,328	9384	27.3	78.9	1.4
37	*Koanophyllon galeottii* (B.L. Rob.) R.M. King & H. Rob.	78	67	481,665	331,545	7205	262,540	128,231	1.5	54.5	26.6
38	*Koanophyllon palmeri* (A. Gray) R.M. King & H. Rob.	93	93	1,131,471	1,447,606	263,411	867,251	17,763	23.3	76.6	1.6
39	*Koanophyllon pittieri* (Klatt) R.M. King & H. Rob.	406	127	333,021	152,754	10,319	113,018	157,329	3.1	33.9	47.2
40	*Lasianthaea fruticosa* (L.) K.M. Becker	878	467	972,478	984,137	227,609	550,550	228,008	23.4	56.6	23.4
41	*Lepidaploa polypleura* (S.F. Blake) H. Rob.	122	121	247,682	36,979	0	30,114	171,540	0.0	12.2	69.3
42	*Lepidospartum squamatum* (A. Gray) A. Gray	334	27	132,657	74,277	0	54,872	43,738	0.0	41.4	33.0
43	*Montanoa frutescens* (Mairet ex DC.) Hemsl.	254	254	565,398	375,408	39,691	259,895	195,086	7.0	46.0	34.5
44	*Montanoa hexagona* B.L. Rob. & Greenm.	50	45	294,889	68,068	0	55,131	184,037	0.0	18.7	62.4
45	*Montanoa karwinskii* DC.	67	67	579,350	565,442	99,152	349,899	115,859	17.1	60.4	20.0
46	*Montanoa leucantha* (Lag.) S.F. Blake	826	826	1,004,151	833,182	63,699	588,544	202,978	6.3	58.6	20.2
47	*Montanoa revealii* H. Rob.	51	51	90,690	55,06	0	4477	69,263	0.0	4.9	76.4
48	*Montanoa speciosa* DC.	93	93	1,142,963	1,139,462	70,460	839,795	74,223	6.2	73.5	6.5
49	*Montanoa tomentosa* Cerv.	714	611	741,882	692,138	59,744	493,406	102,033	8.1	66.5	13.8
50	*Nahuatlea arborescens* (Brandegee) V.A. Funk	64	59	14,120	36,404	17,276	11,072	0	122.4	78.4	0.0
51	*Nahuatlea hypoleuca* (DC.) V.A. Funk	185	179	695,788	568,631	100,275	341,675	196,511	14.4	49.1	28.2
52	*Parthenium fruticosum* Less. ex Schltdl. & Cham.	58	58	268,619	625,789	309,243	174,803	36,718	115.1	65.1	13.7
53	*Parthenium tomentosum* DC.	302	302	619,554	1,277,564	529,608	468,614	19,320	85.5	75.6	3.1
54	*Perymenium grande* Hemsl.	329	176	454,542	224,368	743	181,786	187,402	0.2	40.0	41.2
55	*Peucephyllum schottii* A. Gray	237	15	164,105	182,993	17,727	118,339	3981	10.8	72.1	2.4
56	*Pittocaulon praecox* (Cav.) H. Rob. & Brettell	496	496	592,825	470,700	22,228	353,993	120,575	3.7	59.7	20.3
57	*Pittocaulon velatum* (Greenm.) H. Rob. & Brettell	163	162	509,460	637,622	138,987	369,228	40,493	27.3	72.5	7.9
58	*Pluchea sericea* (Nutt.) Coville	138	46	231,437	225,132	9077	158,609	14,129	3.9	68.5	6.1
59	*Podachaenium eminens* (Lag.) Sch. Bip. ex Sch. Bip.	683	564	619,757	294,788	7450	229,798	270,875	1.2	37.1	43.7
60	*Rensonia salvadorica* S.F. Blake	96	52	126,496	22,543	0	18,456	84,920	0.0	14.6	67.1
61	*Roldana albonervia* (Greenm.) H. Rob. & Brettell	441	441	448,148	251,806	10,237	191,290	168,160	2.3	42.7	37.5
62	*Roldana angulifolia* (DC.) H. Rob. & Brettell	767	767	587,170	258,071	1712	204,876	264,237	0.3	34.9	45.0
63	*Roldana barba-johannis* (DC.) H. Rob. & Brettell	821	817	550,253	228,428	1797	181,755	260,052	0.3	33.0	47.3
64	*Roldana eriophylla* (Greenm.) H. Rob. & Brettell	138	138	500,630	608,966	94,727	396,654	7695	18.9	79.2	1.5
65	*Roldana gentryi* H. Rob. & Brettell	56	56	690,226	475,248	27,835	344,349	200,325	4.0	49.9	29.0
66	*Roldana schaffneri* (Sch. Bip. ex Klatt) H. Rob. & Brettell	264	209	436,186	118,254	1102	94,159	257,213	0.3	21.6	59.0
67	*Rumfordia floribunda* DC.	434	434	376,503	147,338	5695	112,434	190,713	1.5	29.9	50.7
68	*Schistocarpha longiligula* Rydb.	101	74	81,642	7568	494	5677	60,918	0.6	7.0	74.6
69	*Sinclairia glabra* (Hemsl.) Rydb.	495	390	522,077	402,878	53,658	267,867	153,473	10.3	51.3	29.4
70	*Telanthophora cobanensis* (J.M. Coult.) H. Rob. & Brettell	179	156	224,845	41,074	0	33,393	149,708	0.0	14.9	66.6
71	*Telanthophora grandifolia* (Less.) H.Rob. & Brettell	801	602	521,994	217,796	4899	169,326	249,901	0.9	32.4	47.9
72	*Telanthophora uspantanensis* (J.M. Coult.) H. Rob. & Brettell	160	157	375,618	68,252	0	55,137	248,274	0.0	14.7	66.1
73	*Tithonia longiradiata* (Bertol.) S.F. Blake	341	256	187,591	35,500	78	28,755	123,464	0.0	15.3	65.8
74	*Verbesina fastigiata* B.L. Rob. & Greenm.	343	343	526,821	363,759	47,946	242,226	182,200	9.1	46.0	34.6
75	*Verbesina guatemalensis* B.L. Rob. & Greenm.	88	9	144,376	43,261	0	35,374	82,631	0.0	24.5	57.2
76	*Verbesina hypargyrea* B.L. Rob. & Greenm.	58	50	298,605	131,918	22,497	84,905	157,747	7.5	28.4	52.8
77	*Verbesina hypoglauca* Sch. Bip. ex Klatt	149	141	488,252	171,872	12,271	125,163	265,497	2.5	25.6	54.4
78	*Verbesina klattii* B.L. Rob. & Greenm.	155	155	266,302	108,800	9019	77,940	136,101	3.4	29.3	51.1
79	*Verbesina lanata* B.L. Rob. & Greenm.	63	32	155,973	24,034	4323	15,346	111,813	2.8	9.8	71.7
80	*Verbesina montanoifolia* B.L. Rob. & Greenm.	80	80	504,621	339,200	24,377	244,898	157,276	4.8	48.5	31.2
81	*Verbesina oligantha* B.L. Rob.	91	90	437,165	277,890	5494	218,217	135,095	1.3	49.9	30.9
82	*Verbesina oncophora* B.L. Rob. & Seaton	292	292	431,477	196,816	6150	151,754	195,615	1.4	35.2	45.3
83	*Verbesina perymenioides* Sch. Bip. ex Klatt	215	203	463,276	279,876	2385	224,930	151,418	0.5	48.6	32.7
84	*Verbesina turbacensis* Kunth	658	396	482,089	157,618	6000	121,700	269,119	1.2	25.2	55.8
85	*Vernonanthura cordata* (Kunth) H. Rob.	221	220	472,583	382,946	25,378	281,877	99,033	5.4	59.6	21.0
86	*Vernonanthura patens* (Kunth) H. Rob.	748	206	360,749	56,005	786	44,745	248,219	0.2	12.4	68.8
